# Recent advances and challenges of bispecific antibodies in solid tumors

**DOI:** 10.1186/s40164-021-00250-1

**Published:** 2021-12-18

**Authors:** Yuze Wu, Ming Yi, Shuangli Zhu, Haiyong Wang, Kongming Wu

**Affiliations:** 1 Department of Oncology, Tongji Hospital of Tongji Medical College, Huazhong University of Science and Technology, Wuhan, 430030 China; 2 Beijing Anjianxi Medicinal Technology Co., Ltd., No.2 Cuiwei Road, Haidian District, Beijing, 100036 China

**Keywords:** BsAb, Solid tumor, EpCAM, CEA, PSMA, EGFR, HER2

## Abstract

Cancer immunotherapy has made remarkable progress in the past decade. Bispecific antibodies (BsAbs) have acquired much attention as the next generation strategy of antibody-target cancer immunotherapy, which overwhelmingly focus on T cell recruitment and dual receptors blockade. So far, BsAb drugs have been proved clinically effective and approved for the treatment of hematologic malignancies, but no BsAb have been approved in solid tumors. Numerous designed BsAb drugs for solid tumors are now undergoing evaluation in clinical trials. In this review, we will introduce the formats of bispecific antibodies, and then update the latest preclinical studies and clinical trials in solid tumors of BsAbs targeting EpCAM, CEA, PMSA, ErbB family, and so on. Finally, we discuss the BsAb-related adverse effects and the alternative strategy for future study.

## Background

Cancer remains one of the leading contributors to death worldwide [1]. For advanced or metastatic cancers, chemotherapy and radiotherapy are used to be the most effective treatment strategies [2]. Immunotherapy, which moblizes immune system to fight tumor cells [2], has entered the central stage of cancer therapy in recent years. The remarkable triumph of immune checkpoint inhibitors firmly confirmed cancer immunotherapy as the fourth therapeutic option for multiple cancers, such as metastatic melanoma, refractory lung cancer, and advanced breast cancer [3, 4].

Monoclonal antibodies targeting Her2 or EGFR have brought significant response and long-term benefit for the therapy of breast and lung cancer, respectively [5, 6]. Those successes stimulate the development of bispecific antibody [7]. Bispecific antibodies are a rapidly a growing and expanding area of cancer immunotherapy. Initially, blinatumomab was authorized by FDA as a treatment for Philadelphia chromosome-negative acute lymphoblastic leukemia (ALL) in 2014 and recently granted approval for the treatment of minimal residual disease in ALL patients [8, 9]. Although BsAbs have yielded excellent clinical efficacy in hematological malignancies, their therapeutic effect on solid malignancies, which accounted for 90% of all cancers, remains to be established in clinical practice [10]. A major hindering factor for BsAbs in advanced solid tumors is the suppressive tumor microenvironment (TME), which impedes T cell activity and results in immune deficiency [11]. Over the past 10 years, a myriad of BsAbs have been developed, some of which are already in clinical development and many of which are under preclinical testing.

Thus far, BsAbs in cancer immunotherapy have been dominated by T-cell engaging bispecific antibodies (T-BsAbs) [10], which simultaneously binds to tumor-associated antigens (TAA) predominantly expressed on tumor cells and CD3 on T cells, resulting in T-cell activation and triggering target-dependent tumor cell killing. T-BsAbs bridges the interaction of T cells and tumor cells, triggering the activation of the signaling cascade of the T cell receptor (TCR) complex and inducing a transient immunologic synapse between T cells and the tumor cells. Subsequently, perforin and granzymes released from T cells cause the lysis of tumor cells [12]. Notably, the immunologic synapses are not limited by the formation of T cell receptor (TCR) and major histocompatibility complex (MHC) [13]. In comparison to checkpoint inhibitors, T-BsAbs perfectly circumvent the MHC restriction of the TCR to overcome immune escape [14]. This unique approach is a major breakthrough and has been validated in the clinic with the regulatory approval of blinatumomab and catumaxomab [15].

Besides T-BsAbs, the second most widely investigated bispecific antibodies by scientists are those concurrently targeting two epitopes on tumor cells or in the tumor microenvironment (TME) [16]. Unlike the action mechanism of T-BsAbs, BsAbs that target two epitopes on tumor cells function by blocking two mutually related signaling pathways to generate synergistic anti-cancer effect or minimize the drug resistance. For example, MM-111 targets both HER2 and HER3 and has significant clinical effects in patients with non-small cell lung cancer (NSCLC) [17].

## The formats of BsAbs

**Fig. 1:**
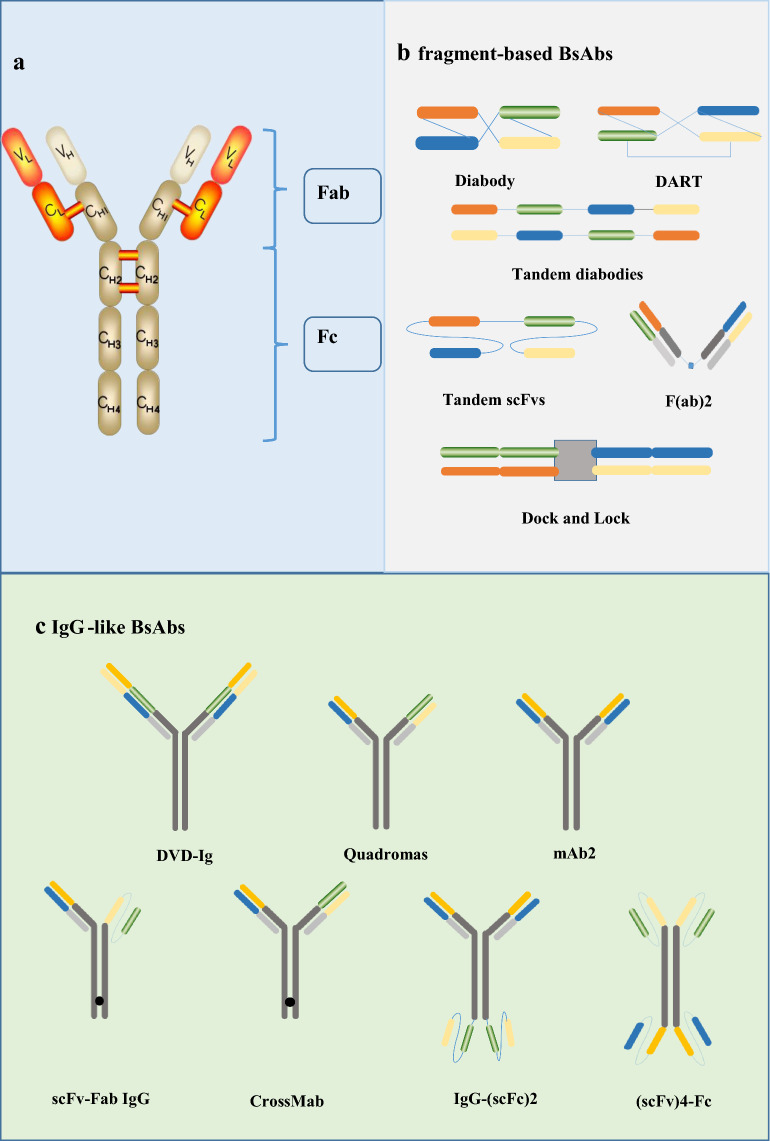
Structure of nature IgG antibody and different formats of bispecific antibodies according to the presence and absence of an Fc region. **a** Structure of nature IgG molecule. **b** fragment-based BsAbs contain Diabody, DART, Tandem diabodies, F(ab)2, Dock and Lock. **c** IgG-like BsAbs mainly include DVD-Ig, Quadromas, mAb2, scFv-Fab IgG, CrossMab, IgG-(scFc)2 and (scFv)4-Fc

The IgG immunoglobulin molecule is composed of two identical heavy chains and light chains, linked together by inter-chain disulfide bonds (Fig. 1a) [18]. IgG antibody can be further subdivided into two distinguished functional segments: fragment of antigen binding (Fab) and the constant fragment (Fc). The Fab unit is the antigen-binding site, determining the antigen specificity. Meanwhile, the Fc fragment is competent to trigger antibody-dependent cell-mediated cytotoxicity (ADCC) as well as complement-dependent cytotoxicity (CDC) [19]. However, nature IgG antibodies cannot simultaneously target the cytotoxic T lymphocytes and the tumor cells, antibodies need to be modified in a variety of approaches to satisfy such a functionality.

Now, the three most commonly used methods to manufacture bispecific antibodies are chemical conjugation, quadroma, and genetic/protein engineering [20]. Over the past decades, the tremendous advances in gene engineering and pharmaceutical techniques have led to an development of BsAbs in varied forms [21]. According to the existence of an Fc region, BsAbs generally can be classified into two major classes: IgG-like molecules (with an Fc domain) and fragment-based molecules (without an Fc domain) (Fig. 1b, c) [22].

IgG-like BsAbs, which mainly contain DVD-Ig, Quadromas and CrossMab, retain antibody-dependent cell-mediated cytotoxicity (ADCC), complement-dependent cytotoxicity (CDC), and antibody-dependent cellular phagocytosis (ADCP) functions mentioned above owing to possessing an Fc unit [23]. In addition, the Fc region of BsAbs can contribute to a longer half-life time for binding the salvage receptor FcRn that plays a key role in reducing the renal clearance rate [24]. Moreover, the Fc region is beneficial to the purification of the bsAbs, as well as improved solubility and stability of BsAbs [25]. In terms of construction, DVD-Ig is manufactured by binding two existing antibodies through a short peptide junction [26]. IgG-scFv is produced through the fusion of scFv with the C-terminus of the IgG light or heavy chain. 

Fragment-based bispecific antibodies generally contain BiTE, Tandem diabodies, DART, diabody [25], consisting of the variable light domains and variable heavy domains of two antibodies or are based on the Fab units of two antibodies, thereby circumventing the chain-association issue [27]. They are smaller size, resulting in enhanced tissue penetration [28]. Thus, it is easier for them to bind to epitopes that are difficult to reach for IgG-like bispecific antibodies. However, the small size of these molecules with the lack of the Fc region causes a particularly rapid renal clearance in vivo. To meet the intended clinical application, fusion to the IgG Fc region or albumin-binding moieties has emerged as a half-life extension strategy [29]. Structurally speaking, diabody is generated by covalently linking two polypeptide chains. Afterwards, Tandem diabodies are constructed by connecting two diabodies with a peptide junction [16].

**Fig. 2:**
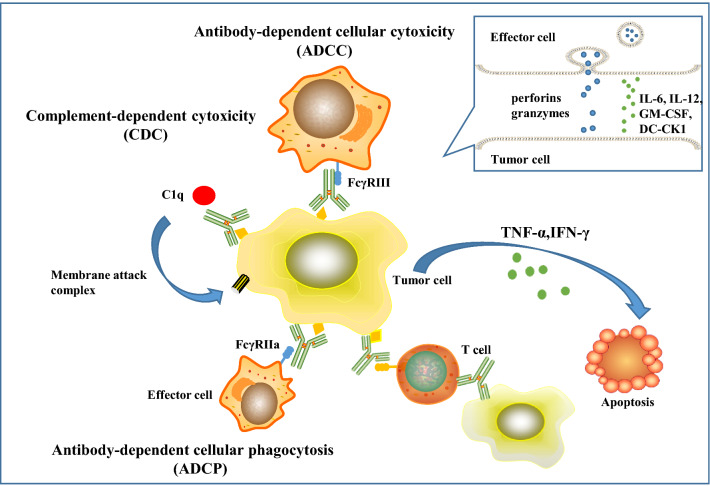
The killing mechanism of TrioMabs (taking Catumaxomab as an example). TrioMabs is a trifunctional BsAb with one arm targeting TAA on tumor cells, another arm targeting CD3 on T cells and the Fc domain binding to Fcγ receptor type I, IIa and III on effector cells such as macrophages, dendritic cells, and NK cells. Once the Fc region of BsAb binds to Fcγ receptor expressed by effector cells or complement component 1q (C1q), these effector cells are activated and release perforins and granzymes from its granules, potentially supporting the destruction of target cells through ADCC, ADCP and CDC, respectively [24]. Additionally, T cells are activated, accompanied by the release of T cell cytokines such as TNF-α and IFN-γ with high levels of proinflammatory cytokines such as IL-6, IL-12, GM-CSF, and DC-CK1 [34, 35]

More specifically, TrioMabs and BiTEs are formats at the frontier of clinical trials [30]. Two BsAbs—catumaxomab (TrioMab) and blinatumomab (BiTE)—have been approved for the treatment of cancer patients [31]. TrioMab is designed to eliminate tumor cells by its trifunctional mode of action [32]. Taking as an example, catumaxomab is an intact trifunctional BsAb with one arm targeting human epithelial cell adhesion molecule (EpCAM) on tumor cells, one arm targeting CD3 on T cells and the Fc region binding to Fcγ receptor type I, IIa and III on effector cells such as macrophages, nature killer cells and dendritic cells [32]. Once the Fc region of BsAb binds to Fcγ receptor or complement component 1q, these effector cells are activated and release perforins and granzymes from their granules [33], accelerating the destruction of tumor cells through ADCC, ADCP, and CDC, respectively [24]. Additionally, activated T cells release abundant amounts of TNF-α and IFN-γ cytokines, accompanied by high levels of cytokines such as IL-6, IL-12, GM-CSF, and DC-CK1 [34, 35] (Fig. 2). Due to this triangular binding capability, TrioMabs can stimulate not only the innate immune system but also the adaptive immune system [36]. The format of BiTE contains the variable light chains and variable heavy chains of two antibodies linked by a flexible linker [28]. It is reported that BiTE was far superior to other conventional bispecific antibody formats because BiTE exhibited better anti-tumor activity, less dosage, and lower production costs [37].

To date, more than 180 BsAbs are in preclinical development and over 50 BsAbs have been investigated in clinical trials. Global clinical trials of BsAbs are focused on Phase I, Phase I/II and Phase II, while Phase III clinical trials are still rare [38]. In this review, we will mainly summarize the relevant clinical studies of BsAb in solid tumors with various targeting antigens (Table 1) and discuss the BsAb-related side effects in clinical application.

**Table 1:** Recent advances and challenges of bispecific antibodies in solid tumor

Targets	BsAb	Status	Phase	NCT Number	Conditions
EpCAM/CD3	Catumaxomab	Completed	II	NCT01065246	Malignant Ascites Due to Epithelial Carcinoma
		Completed	II	NCT00377429	Ovarian Cancer
		Completed	II	NCT00464893	Gastric Cancer, Gastric Adenocarcinoma
		Completed	II	NCT01246440	Ovarian Cancer
		Completed	II	NCT01504256	Gastric Adenocarcinoma With Peritoneal Carcinomatosis
		Completed	II	NCT00326885	Malignant Ascites
		Completed	II	NCT01815528	Recurrent Epithelial Ovarian Cancer
		Completed	III	NCT00822809	Cancer, Neoplasms
		Completed	II	NCT00563836	Ovarian Cancer, Epithelial Ovarian Cancer
		Completed	II/III	NCT00836654	EpCAM Positive Cancer
		Completed	II	NCT00352833	Gastric Cancer, Gastric Adenocarcinoma
		Completed	II	NCT00189345	Ovarian Cancer, Fallopian Tube Neoplasms, Peritoneal Neoplasms
		Not recruiting	I/II	NCT04799847	Bladder Cancer
		Recruiting	I	NCT04819399	Urinary Bladder Neoplasms
		Recruiting	III	NCT04222114	Stomach Neoplasms
		Terminated	I	NCT01320020	Epithelial Cancer
		Terminated	II	NCT01784900	Gastric Peritoneal Carcinomatosis
	MT110 (AMG110)	Completed	I	NCT00635596	Solid Tumors
CEA/CD3	MT111/AMG211/MEDI-565	Completed	I	NCT01284231	Gastrointestinal Adenocarcinomas
		Terminated	I	NCT02291614	Gastrointestinal Cancer
	RO6958688 (RG7802)	Completed	I	NCT02650713	Solid Tumors
		Completed	I	NCT02324257	Solid Tumors
		Recruiting	III	NCT03337698	NSCLC
CEA/HSG	TF2 (IMP288)	Completed	III	NCT01221675	SCLC, CEA-expressing NSCLC
		Withdrawn	I	NCT01273402	Metastatic Colorectal Cancer
		Completed	II	NCT02587247	Metastatic Colorectal Cancer
		Completed	I	NCT00860860	Colorectal Neoplasms
		Unknown	III	NCT02300922	Metastatic Colorectal Cancer
PSMA/CD3	Pasotuxizumab (BAY 2010112, MT112, AMG 212)	Completed	I	NCT01723475	Prostatic Neoplasms
	Acapatamab (AMG160)	Recruiting	I	NCT04822298	Non-Small Cell Lung Cancer
		Recruiting	I	NCT03792841	Metastatic Castration-resistant Prostate Cancer
		Recruiting	III	NCT04631601	Metastatic Castration-resistant Prostate Cancer
	HPN424	Recruiting	III	NCT03577028	Advanced Prostate Cancer
	MOR209/ES414	Completed	I	NCT02262910	Metastatic Castration-resistant Prostate Cancer
	CC-1	Recruiting	I	NCT04104607	Metastatic Castration-resistant Prostate Cancer
		Not yet recruiting	III	NCT04496674	Lung Cancer Squamous Cell
EGFR/MET	Amivantamab (JNJ-61186372)	Approved for marketing		NCT04599712	Metastatic NSCLC
		Recruiting	II	NCT04945733	Stomach Neoplasms, Esophageal Neoplasm
		Recruiting	II	NCT04965090	Metastatic NSCLC
		Recruiting	III	NCT04538664	Metastatic NSCLC
		Not recruiting	III	NCT04988295	NSCLC
		Recruiting	I	NCT04606381	Advanced Solid Malignancies
		Recruiting	III	NCT04487080	NSCLC
		Recruiting	I	NCT02609776	NSCLC
		Recruiting	I	NCT04077463	NSCLC
HER2/CD3	Ertumaxomab	Terminated	III	NCT01569412	HER2/Neu Positive Advanced Solid Tumors
		Terminated	II	NCT00351858	Metastatic Breast Cancer
		Terminated	II	NCT00522457	Metastatic Breast Cancer
		Terminated	II	NCT00452140	Metastatic Breast Cancer
HER2/HER3	MM111	Completed	I	NCT01097460	Breast Neoplasms
		Completed	I	NCT00911898	HER2 Amplified Solid Tumors
		Completed	I	NCT01304784	HER2 Amplified Solid Tumors
		Terminated	II	NCT01774851	HER2 Positive Carcinomas of the Distal Esophagus, Gastroesophageal Junction and Stomach
HER2/HER3	Zenocutuzumab (MCLA-128)	Recruiting	III	NCT02912949	Solid Tumors Harboring NRG1 Fusion
GPC3/CD3	ERY974	Completed	I	NCT02748837	Solid Tumors
		Recruiting	I	NCT05022927	Hepatocellular Carcinoma
PD1/PDL1	LY3434172	Completed	I	NCT03936959	Advanced Cancer
PDL1/TIM3	LY3415244	Terminated	I	NCT03752177	Advanced Cancer
PD-1/CTLA4	MEDI5752	Active	I	NCT03819465	Metastatic NSCLC
		Recruiting	I	NCT03530397	Advanced Renal Cell Carcinoma
		Recruiting	I	NCT04522323	Advanced Solid Tumors
DLL4/VEGF	Navicixizumab (OMP-305B83)	Not recruiting	III	NCT05043402	Ovarian Cancer, Fallopian Tube Cancer, Primary Peritoneal Carcinoma
		Completed	I	NCT02298387	Advanced Solid Tumor Malignancies
		Completed	I	NCT03030287	Ovarian, Peritoneal or Fallopian Tube Cancer
		Terminated	I	NCT03035253	Metastatic Colorectal Cancer
DLL4/VEGF	ABL001 (NOV1501)	Recruiting	III	NCT04492033	Advanced Solid Tumors
		Completed	I	NCT03292783	Advanced Solid Tumors
CD3/GPA33	MGD007	Completed	I	NCT02248805	Colorectal Carcinoma
		Active	III	NCT03531632	Metastatic Colorectal Cancer
CD64/EGFR	MDX447	Completed	I	NCT00005813	Brain and Central Nervous System Tumors

*EpCAM* epithelial cell adhesion molecule, *CEA* carcinoembryonic antigen, *HSG* human serum albumin, SCLC small cell lung cancer, *NSCLC* non-small cell lung cancer, *PSMA* prostate-specific membrane antigen, *NRG1* neuregulin 1, *GPC3* glypican 3, *HCC* hepatocellular carcinoma, *PD-1* programmed cell death protein 1, *PD-L1* programmed cell death ligand 1, *TIM3* T cell immunoglobulin and mucin domain-containing protein 3, *CTLA4* cytotoxic T lymphocyte associate protein-4, *DLL4* delta-like ligand 4, *VEGF* vascular endothelial growth factor, *EGFR* epidermal growth factor receptor

## Targeting antigens

Targets that are frequently pursued by multiple commercial companies include EpCAM in NSCLC, HER2 in advanced breast cancers, prostate-specific membrane antigen (PSMA) in castration-resistant prostate cancers, and carcinoembryonic antigen (CEA) in colorectal cancers [10]. Other notable targets that have recently been in preclinical development is glypican 3 (GPC3) in liver cancers, as well as GPA33 in colorectal cancers Table 1 [39].

### EpCAM

EpCAM is a conserved type I transmembrane protein, the extracellular domain of which contains a signal peptide, an EGF-like, cysteine-rich domain, and a thyroglobulin-like domain followed by a cysteine-poor region [40]. EpCAM is related to several signaling pathways including the intramembrane protein hydrolysis (RIP)-mediated signaling and the activation of Wnt signaling to impact on tumor cell proliferation [41]. Today, EpCAM is reckoned as a marker highly expressed in carcinomas and extremely correlated with poor prognosis [42]. A large retrospective study using tissue microarrays showed that high level of EpCAM expression was found in 94.1% of patients with lung, colon, and prostate cancers and observed a significant reverse correlation between EpCAM expression and survival time [43]. Therefore, EpCAM represents an attractive therapeutic target for bispecific antibodies.

Catumaxomab (TrioMabs) was authorized by the European Union for the intraperitoneal treatment of patients with malignant ascites produced by epithelial carcinomas [44]. Preclinical trials have demonstrated lysis of tumor cells with catumaxomab in vitro and in vivo [45]. Based on the results of clinical trials below, the intraperitoneal administration of catumaxomab has an extraordinary clinical efficacy [44]. A prospective study enrolled eight patients with malignant ascites because of peritoneal carcinomas treated with catumaxomab. The accumulation of ascites disappeared in all patients and no severe AEs were observed [46]. A phaseIIstudy included 45 patients with refractory epithelial ovarian cancer demonstrated that the escalating dose regimen of catumaxomab was safer and higher efficacy index than the constant low dose regimen [47]. In another two-arm phase II/III trial, investigators randomly assigned 258 patients with malignant ascites to two groups: catumaxomab plus paracentesis and paracentesis alone. Patients who received catumaxomab treatment had reduced ascites, as well as longer puncture-free survival than control patients did. Moreover, adverse events were manageable and generally reversible [48]. To assess the safety and acceptability of intravenous administration with catumaxomab, a phase I study was conducted in patients with NSCLC. The maximum tolerated dose (MTD) was demonstrated to be 5 µg [49]. Another phase I study enrolled 16 patients who had known EpCAM expression. The study results showed the MTD of intravenously injected catumaxomab was 7 µg. The most common adverse events (AEs) were chills and pyrexias and the major toxicities were cytokine release-related symptoms and hepatotoxicity. As the first patient who took 10 µg developed a fatal liver failure, the study was forced to terminate [50]. More recently, a phase II study demonstrated that the combination of catumaxomab and chemotherapy was feasible and bearable in patients with gastric cancer [51]. However, catumaxomab is not being manufactured anymore on account of toxicity issues.

MT110 (solitomab, AMG110) is a BiTE construct targeting EpCAM. MT110 has been shown to induce synergistic stimulation of CD4- and CD8-positive T cells and to reactivate tumor-resident T cells to eradicate tumor cells [52, 53]. Then, an in vitro study indicated existence of a significant therapeutic window for MT110 in mice [54]. In a phase I dose-escalation clinical trial, 65 patients with recurrent solid cancers who could not receive standard therapy were given serial intravenous infusion of MT110. The study demonstrated that the MTD was 24 µg. Diarrhea, increased liver parameters, and elevated lipase were observed as common AEs [55].

### CEA

CEA is also known as carcinoembryonic antigen-associated cell adhesion molecule 5 (CEACAM5), which anchors to the cell surface by glycosylphosphatidylinositol [56]. CEA is overexpressed in many cancers and associated with adhesion and invasion [57]. Therefore, CEA has been served as a prognostic factor for colorectal carcinoma [58]. Then, several studies have reported that circulating CEA levels are a valuable supportive diagnostic tool for both non-small cell lung cancer (NSCLC) and SCLC [59, 60]. Indeed, CEACAM5 is now considered as clinically effective biomarker and prospective target in various solid malignancies, the majority of which are colorectal cancers.

MEDI-565 (MT111, AMG211), a bispecific T-cell engager antibody, concurrently targets human CEA and the CD3 [61]. A preclinical study proved that MEDI-565 activated T cells to eliminate cancer cells not only in vivo but also in vitro [62]. To explore MEDI-565 biodistribution, a viability study was performed in 9 patients with terminal gastrointestinal adenocarcinomas. This study clearly showed the drug highly accumulated in CD3-rich lymphoid tissues and significantly taken up by tumor tissues [63]. NCT01284231 is a phase I dose-escalation study that included 39 patients with gastric or intestinal tumors. Like other BiTEs, MEDI-565 is cleared with a much shorter half-life due to the absence of the Fc segment. The MTD of MEDI-565, as the primary objective, was 5 mg. Median overall survival (OS) time was 5.5 months and 11 patients exhibited stable disease (SD). Nausea, abdominal pain, vomiting and fatigue were considered as the most common AEs [64].

RO6958688 (RG7802) is a new IgG-based T-BsAb constructed by CrossMab technology, containing two CEA-binding sites, one CD3-binding site and a silenced Fc region. In preclinical models, RO6958688 increased T-cell infiltration within the tumor and created a highly inflammatory tumor microenvironment, exhibiting extremely strong anti-tumor capacity [65]. Study by Lehmann et al. suggested that RO6958688 speeded up cancer cell lysis by enabling the linkage of more than one T cells with a single tumor cell [66]. A dose-escalation Phase I clinical study of RO6958688 in participants with advanced or metastatic CEA positive solid malignancies (NCT02324257) was completed. 80 patients received dosage levels ranging from 0.05 mg to 600 mg. The study observed reliable antitumor efficacy of RO6958688 monotherapy. Pyrexia, infusion-related reaction and diarrhea were regarded as the most common AEs. Subsequently, NCT02650713, an extended Phase Ib clinical study of RO6958688 in addition with anti-PD-L1 antibody, was initiated on 38 patients. Two patients experienced partial responses (PR) to treatment whereas five patients developed stable disease. More importantly, the anti-PD-L1 antibody, atezolizumab, appeared to enhance the anti-tumor activity of RO6958688, with a manageable safety profile [67].

### PMSA

PSMA, also known as glutamate carboxypeptidase II, is a type II transmembrane protein with folate hydrolase activity produced by prostatic epithelium cells [68]. It is significantly overexpressed in androgen-independent prostate cancers while highly restricted in extraprostatic tissues [69]. Immunohistochemistry study revealed that the degree of PSMA expression correlated with the pathological grade, disease progression and recurrence [70–73]. Those characteristics make PMSA an excellent target for bispecific antibodies.

Pasotuxizumab (BAY 2010112) is a T-BsAb targeting PSMA. In a multicenter, dose-escalation study (NCT01723475), 16 patients with metastatic castration-resistance prostate cancer (mCRPC) who received continuous intravenous infusion of Pasotuxizumab were enrolled into five dosing cohorts. There were two long-term responders in the dose-escalation and one of them showed a CR. Fever, chills, and fatigue were reported as major AEs [74, 75].

HPN424, derived from the TriTAC platform, functions through three binding domains: two arms binding PSMA and CD3 separately and an attached albumin for half-life extension. An open-label, Phase 1/2a study of HPN424 monotherapy in patients with advanced prostate cancer refractory to androgen therapy (NCT03577028) is ongoing. 27 patients were dosed in eight cohorts. 11 out of 19 participants observed reduction in circulating tumor cells (CTCs). Six patients were found to have a decline in PSA from baseline. No dose-limiting toxicity (DLTs) had been observed and all AEs were transient and manageable [76].

Similarly, AMG 160 is PSMA-targeted T-BsAb with a prolonged half-life [77]. A preclinical model of mCRPC highlighted its potent anti-tumor efficacy both in vitro and in vivo and its acceptable safety profile in nonhuman primates [78]. Those data supported the upcoming clinical assessment of AMG 160 in patients with mCRPC (NCT03792841). More recently, preliminary results from the dose exploration section of the ongoing phase I study were reported. 32 patients were administered AMG160. MTD has not been reached. Among 18 patients, responses included one confirmed partial response (PR), five stable disease (SD) and five progressive disease (PD). In addition, Six participants received the combination of AMG 160 and pembrolizumab and no DLTs were reported [79].

### ErbB family

ErbB family includes four closely relate transmembrane tyrosine kinase receptors named EGFR (HER1), HER2, HER3, and HER4, which share a highly conserved extracellular ligand-binding domain, a transmembrane junction, and an intracellular segment with a tyrosine kinase domain (except for HER3) [80]. Several ligands that bind HER1, HER3 and HER4 have been identified, such as transforming growth factor α (TGFα), epidermal growth factor (EGF), and neuregulins (NRG) [81]. Unlike other members, no natural ligand has been identified for HER2. It functions by forming homodimers or heterodimers with EGFR, HER3 [82]. Notably, the intracellular component of HER3, rather than tyrosine kinase domain, contains a plurality of binding sites for phosphatidylinositol 3-kinase (PI3K) signal pathway [17].

HER2 is a transmembrane glycoprotein overexpressed in breast cancer, bladder cancers, cervix cancers, gallbladder cancers, endometrium cancers and ovarian cancers. Overexpression of HER2 receptors has been reported to be highly associated with poor prognosis and related to reduced PFS and OS as well [83].

Ertumaxomab is a trifunctional bispecific antibody targeting HER2 and CD3 with a third binding to activating accessory cells such as macrophages and dendritic cells [84]. Ertumaxomab was reported to be capable of mediating the elimination of cancer cells with overexpressed HER2 even at low levels, which was accompanied by a Th1-based cytokine release [85]. In a multicenter phase I clinical trial, 15 out of 17 enrolled patients with HER2-positive metastatic breast cancer completed the study. MTD was demonstrated to be 100 μg/kg. Among 11 evaluable patients, 5 participants displayed anti-tumor activity including two PR, one CR, and two SD [86]. Moreover, in order to evaluate the safety and effectiveness of Ertumaxomab, another phase I clinical trials was performed in patients with metastatic breast cancer and HER2-positive advanced solid tumors. MTD was not reached. DLT was not detected. Three patients were seen to have clinical response to Ertumaxomab with one PR and two SD [87, 88].

EGFR is a transmembrane glycoprotein consisting of an extracellular ligand-binding domain and an intracellular protein tyrosine kinase domain that is linked by a small transmembrane anchoring region [89]. EGFR activates multiple intracellular signaling pathways, such as PLC-γ-PKC, Ras-Raf-MEK, PI3K-Akt-mTOR, and JAK2-STAT3 [90], which ultimately affect cell proliferation, survival, motility, and adhesion. Excessive activation of EGFR signaling pathway is detected in various advanced solid tumors, including NSCLC, breast cancer, colorectal cancer, and ovarian cancer. Therefore, targeting ErbB signaling by tyrosine kinase inhibitors (TKIs) is extensively developed.

In the past two decades, treatment of NSCLC harboring EGFR mutation with TKIs has achieved great success [91]. Unfortunately, the majority of patients eventually relapse or become drug resistant [81], because there is considerable heterodimerization and crosstalk between all four members of the ErbB family [82]. Therefore, BsAbs that simultaneously block two or more RTK signaling pathways can diminish the probability of this evasion mechanism and thus augment efficacy. Previous preclinical and clinical studies have suggested that *MET* gene amplification is an important mechanism underlying acquired resistance to three generations of EGFR-TKIs [92]. Accordingly, simultaneous blockade of MET and EGFR theoretically should be considered for patients with resistant NSCLC carrying MET amplification.

Amivantamab (JNJ-61186372) is an anti-EGFR and anti-MET bispecific antibody with enhanced Fc function [93]. In multiple preclinical models, potent in vivo antitumor efficacy of Amivantamab was observed [94]. Further analysis demonstrated monocytes and/or macrophages were required for EGFR/MET down-modulation and in vivo antitumor efficacy [95]. Amivantamab was demonstrated to employ three mechanisms to inhibit tumors with diverse EGFR mutations and complete and durable tumor regression was noticed in the combination treatment of amivantamab and a third-generation EGFR-TKI (AZD9291) [96]. An ongoing phase 1 study of amivantamab consists of two phases, dose-escalation and dose-expansion, enrolling patients with EGFR and MET mutations. Amivantamab was intravenously injected to participants. This therapeutic schedule exhibited a controlled safety profile without DLTs observed. Rash, infusion-related reaction and paronychia were considered as the most common AEs [97]. More recently, 50 enrolled patients with exon20ins mutations were treated with amivantamab. Among the 39 assessable patients, the overall response rate was 36%. The clinical benefit rate was 67% and the median PFS was 8.3 months [98]. Therefore, The FDA granted accelerated approval to amivantamab, the first agent directed against two tumor antigens [93, 99].

MM-111, a novel anti-HER2 and anti-HER3 BsAb with modified human serum albumin (HSA), specifically targets the HER2/HER3 heterodimer and effectively inhibits HER3 corresponding downstream signaling pathways [100]. A previous study demonstrated that MM-111 plus trastuzumab would synergistically inhibit tumor growth achieving 25% greater cell growth inhibition than the effects of the individual treatments [101]. NCT00911898 was a completed PhaseIclinical trial of MM-111 monotherapy in patients with HER2 amplified solid tumors. 20 enrolled participants were administered of MM-111 weekly via IV. MTD was not attained as no patients experienced a dose-limiting toxicity. In another PhaseIclinical trial of MM-111 in addition with herceptin (NCT01097460), two of 16 participants with breast neoplasms had serious adverse events. Furthermore, a dose ascending study of MM-111 with five different combined remedies (NCT01304784) was conducted to determine the safety of MM-111 with diverse combination regimens. In addition, a PhaseIIstudy of MM-111 and paclitaxel with trastuzumab (NCT01774851) enrolled approximately 120 patients who were randomly assigned to the experimental or control groups. PFS were reported as 30 months. However, this study was early terminated due to lack of efficacy.

Zenocutuzumab (MCLA-128), an IgG-like bispecific antibody, concurrently targets HER2 and HER3. Served as the ligand of the receptor HER3, NRG1 binding to HER3 results in heterodimerization of the HER3 and HER2 and activation of downstream signaling including the ERK and PI3K–AKT pathways, which play a significant role in tumor cell multiplication and tumor expansion. Thus, MCLA-128 could be a potent drug for NRG1-fused tumors. Preclinical testing of MCLA-128 demonstrated its effectiveness in ovarian and breast cancer models harboring various NRG1 fusions [102]. Another preclinical study had evaluated pharmacokinetics and pharmacodynamics of MCLA-128 to predict a safe starting dose for the following clinical study [103]. In order to evaluate the security, tolerability and anti-tumor efficacy of MCLA-128, a Phase I/II dose-escalating clinical trial in patients with solid cancers carrying an NRG1 fusion is initiated (NCT02912949).

### GPC3

GPC3 belongs to the glypican family of heparan-sulfate proteoglycans and is attached to the cell membrane via a glycosylphosphatidylinositol (GPI) anchor [104]. GPC family comprises six members, GPC1 to GPC6, and the abnormal expression of GPC stimulates cancer cell proliferation and tumor progression by modulating Wnt, hedgehog and bone morphogenetic proteins. Importantly, GPC3 is highly represented in over 70% of hepatocellular carcinoma (HCC), which is one of the most prevalent cancers worldwide [105]. Moreover, previous studies have demonstrated a strong correlation between high levels of GPC3 expression and poor prognosis of HCC [106, 107]. Therefore, GPC3 becomes a promising target for antibody-based immunotherapies for HCC.

ERY974, a completely humanized IgG structured antibody, directs T cells to non-immunogenic tumors using expression of GPC3 to confer tumor specificity [108]. A study in 2020 indicated that ERY974 was effective in suppressing GPC3-expressing tumor growth [109]. In a reconstituted mouse model, ERY974 was found to have excellent antitumor activity against diverse types of GPC3-expressing tumor cells. More importantly, ERY974 greatly increased the number of inflammatory cells in the tumor microenvironment, turning it into a highly inflamed microenvironment [39]. These preclinical results facilitated the initiation of clinical trials of ERY974 for solid tumors. A multicenter Phase 1 study of ERY974, consisting of a dose escalation and a cohort expansion, was recently completed. However, there is no results posted in ClinicalTrials.gov so far.

### Immune checkpoint

Immune checkpoints have immunosuppressive regulatory effects, including programmed cell death protein 1 (PD-1) and its ligand programmed cell death protein ligand 1 (PD-L1), and cytotoxic T lymphocyte-associated protein 4 (CTLA-4). When immune checkpoints are overexpressed, the immune homeostasis is disrupted and T-cell immune responses were suppressed, leading to immune evasion of tumor cells [110]. When compared to PD-1 and/or PD-L1 monotherapy, previous preclinical studies have described that BsAbs simultaneously targeting PD-1 and PD-L1 had significant activation of T cells, thus supporting the evaluation of BsAbs targeting immune checkpoints.

LY3434172, a human IgG-like bispecific antibody with a silent Fc region targeting PD-1 and PD-L1. One arm of LY3434172 blocks the binding of PD-1 to PD-L1 and PD-L2, while the other arm blocks the binding of PD-L1 to PD-1 and the agonist receptor, CD80. It has been demonstrated that LY3434172 resulted in robust antitumor activity at doses substantially lower than either parent antibody or their combination in established human xenograft models [110]. A phase 1 study (NCT03936959) of LY3434172 monotherapy in metastatic solid cancers was completed. So far, there was no results posted.

LY3415244 is a bispecific antibody simultaneously targeting TIM-3 and PD-L1. In a phase I clinical trial conducted in patients with advanced solid tumors, LY3415244 was administered intravenously to 12 patients with advanced solid tumors. However, treatment-emergent antidrug antibodies (ADA) were detected in every participant and the study had to be terminated [111].

In addition, there are multiple rationally designed dual-targeted immune checkpoint bispecific molecules aiming to provide superior activity. Clinical trials are testing a number of immune checkpoint bispecific antibodies, including PD-1xCTLA-4, PD-1xLAG3 (lymphocyte activation gene-3) and PD-L1xTGFb [112–114].

### DLL4 and VEGF

Delta-like ligand 4 (DLL4) is a member of Notch receptor ligand family, which plays a central role both in the formation of the vasculature and neo-vascular formation. During neovascularization, DLL4 coordinates with vascular endothelial growth factor (VEGF) to regulate the formation of new tip cells, which ultimately promote vascular branching through various cell migration steps [115]. However, previous studies have shown that blocking DLL4 signaling pathway in the blood vessels resulted in upregulation of VEGF and overproliferation of endothelial cells. Therefore, simultaneously targeting DLL4 and VEGF would possibly bypass this resistance mechanism and augment the anti-tumor effects of DLL4 inhibitors. The biologic effects of double inhibition DLL4 and VEGF were assessed in ovarian cancer models. The results indicated simultaneously targeting VEGF and DLL4 displayed a superior antitumor efficiency compared with either monotherapy [116].

Navicixizumab (OMP-305B83) is an IgG-like BsAb that suppresses tumor vascularization by blocking both DLL4 and VEGF. It was examined in several preclinical human tumor xenograft models, including colon, ovarian and other cancers and all studies demonstrated significant anti-tumor activity. A phase I dose-escalation study (NCT02298387) enrolled 66 patients with relapsed solid cancers. Among them, four patients had PR and 17 patients had SD. Of note, nine participants had a decrease in the size of tumor lesions. The MTD was not determined. The most frequent navicixizumab-related AEs were hypertension, fatigue, headache and pulmonary hypertension [117]. Then, navicixizumab had been further investigated in combination with paclitaxel in platinum-resistant ovarian cancer patients in a Phase 1b study (NCT03030287). By 2020, 44 patients had been included and the overall response rate (ORR) of this combination was 43% including 1 CR and 18 PR [118].

### Others

Apart from those targets mentioned above undergoing clinical trials, multiple targets in preclinical development have shown very encouraging and impressive effectiveness in anti-tumor activity. Tebentafusp is a bispecific protein that can redirect T cells to specific glycoprotein 100-positive cells. In a phase III clinical trial, 378 patients with metastatic uveal melanoma were randomized to the tebentafusp group or the control group. OS at 1 year and PFS were significantly higher in the tebentafusp group than in the control group. Rash, pyrexia, and pruritus were considered as the most common AEs. No treatment-related deaths were reported [119]. In addition, GPA33 was a selected cancer antigen as it is commonly found in both primary and metastatic colorectal carcinomas. MGD007, a fragment-based BsAb aimed at redirecting T cells to GPA33-positive tumor cells, displayed the ability to inhibit tumor proliferation at low doses in a xenograft model [120].

## Adverse effects of BsAb treatment

In 2014, FDA approved Blinatumomab for relapsed or refractory ALL. Along with the remarkable efficacy of antibody-based immunotherapies in clinical applications, there is a growing awareness of their inherent and potentially life-threatening adverse effects. In a phase I/II study of Blinatumomab in patients with ALL, 3 patients and 1 patient had cytokine release syndrome (CRS) of grade 3 and 4, respectively [121]. Similarly, another phaseIIstudy reported three patients had grade 3 cytokine release syndrome [122]. Previous studies on Blinatumomab suggested that the CRS is the most noteworthy AEs [123].

CRS is an overshooting systemic inflammatory response with a wild range of symptoms varying from mild, flu-like symptoms to severe anaphylactic shock with a life-threatening presentation. CRS-related laboratory abnormalities include cytopenia, elevated liver enzymes, dysregulation of coagulation parameters and elevated ferritin, etc. When patients receive T-BsAb treatment, multiple activated T cells release large amounts of IFN-γ, inducing the activation of other immune cells (macrophages). Then, activated macrophages subsequently produce excessive amounts of inflammatory cytokines such as IL-6, TNF-α, and IL-10, which eventually develop into CRS. Elevations of IL-6, IL-10, and IFN-γ are most frequently found in the serum of patients with CRS [124].

Pretreatment with corticosteroids is the golden standard to reduce CRS.A recent study demonstrated that an optimized dosing regimen could significantly alleviate CRS [125]. At present, the most efficient approach for controlling CRS is a combination of dose escalation and pretreatment with corticosteroid. Apart from the above, there are still many approaches to reduce CRS being tested.

Emerging evidence indicates that IL-6 is at the core of inflammatory cytokines in CRS [126]. IL-6, mainly released by macrophages, is a multivariable cytokine with an essential function in the modulation of immune response and inflammation [127]. Theoretically, blocking IL-6 actions could attenuate CRS toxicity without compromising T cell-mediated antitumor activity. Therefore, a patient who was prospectively monitored during Blinatumomab treatment period developed CRS with fever, respiratory failure and circulatory failure. Fortunately, the participant rapidly recovered after injected tolimumab, an anti-IL-6 receptor antagonist [128]. This case report provides a new insight into mitigating the adverse effect, CRS, causing by BsAbs retaining Fc domain.

## Conclusion

With the biotechnology advance and new target identification, therapeutic bispecific antibodies are a hot field for drug development. So far, two bispecific antibody drugs have gained regulatory permission and are currently on the market. Although no bispecific antibody for solid tumors has been licensed for clinical application, over 100 BsAbs for a wide variety of solid malignancies have achieved promising preclinical results and are in the stages of prospective clinical trial.

BsAbs come in various formats, which influence manufacturing, valency, Fc-mediated effector functions, and in vivo half-life. Selecting right two targets to achieve synergistically therapeutic effect than monoclonal antibody is challenging. Obviously, choosing the right format of BsAbs and selecting the rational combination of targeting antigens is the key to successful BsAbs.

In conclusion, multiple BsAbs provide exciting preclinical response and promising clinical outcomes. We anticipate that the continuing optimization of antibody structure and rational combination of targeting molecule will eventually produce BsAbs for the cancer immunotherapy of solid cancers.

## Data Availability

Not applicable.

## Abbreviations

BsAb: Bispecific antibody
T-BsAb: T-cell engaging bispecific antibodies
BiTE: Bispecific T-cell engager
DVD-Ig: Dual variable domain immunoglobulin
ALL: Acute lymphoblastic leukemia
ADCC: Antibody-dependent cell-mediated cytotoxicity
CDC: Complement-dependent cytotoxicity
ADCP: Antibody-dependent cellular phagocytosis
NSCLC: Non-small cell lung cancer
TME: Tumor microenvironment
TCR: T cell receptor
MHC: Major histocompatibility complex
CTL: Cytotoxic T lymphocyte
EpCAM: Epithelial cell adhesion molecule
RIP: Intramembrane protein hydrolysis
MTD: Maximum tolerated dose
AEs: Adverse events
CEA: Carcinoembryonic antigen
CEACAM5: Carcinoembryonic antigen-associated cell adhesion molecule 5
OS: Overall survival
PMSA: Prostate-specific membrane antigen
mCRPC: Metastatic castration-resistant prostate cancer
CR: Complete regression
PR: Partial response
SD: Stable disease
PD: Progressive disease
ORR: Overall response rate
DLT: Dose limiting toxicity
CTL: Circulating tumor cell
EGFR: Epidermal growth factor receptor
TGFα: Transforming growth factor α
TKI: Tyrosine kinase inhibitors
PTK: Receptor tyrosine kinase
IGF: Insulin-like growth
HSA: Human serum albumin
GPC3: Glypican 3
VEGF: Vascular endothelial growth factor
DLL4: Delta-like ligand 4
PD-1: Programmed cell death protein 1
PD-L1: Programmed cell death ligand 1
TIM-3: T cell immunoglobulin and mucin domain-containing protein 3
ADA: Antidrug antibodies
LAG3: Lymphocyte activation gene-3
CRS: Cytokine release syndrome
HCC: Hepatocellular carcinoma
PFS: Progression-free survival

## References

[1] Binder M, Roberts C, Spencer N, Antoine D, Cartwright C (2014). On the antiquity of cancer: evidence for metastatic carcinoma in a young man from ancient Nubia (c. 1200BC). PLoS ONE.

[2] Wu Z, Cheung NV (2018). T cell engaging bispecific antibody (T-BsAb): from technology to therapeutics. Pharmacol Ther.

[3] Alsaab HO, Sau S, Alzhrani R, Tatiparti K, Bhise K, Kashaw SK (2017). PD-1 and PD-L1 checkpoint signaling inhibition for cancer immunotherapy: mechanism, combinations, and clinical outcome. Front Pharmacol.

[4] Zahavi D, AlDeghaither D, O'Connell A, Weiner LM (2018). Enhancing antibody-dependent cell-mediated cytotoxicity: a strategy for improving antibody-based immunotherapy. Antib Ther.

[5] Bradley JD, Hu C, Komaki RR, Masters GA, Blumenschein GR, Schild SE (2020). Long-term results of NRG oncology RTOG 0617: standard- versus high-dose chemoradiotherapy with or without cetuximab for unresectable stage III non-small-cell lung cancer. J Clin Oncol.

[6] Yu S, Liu Q, Han X, Qin S, Zhao W, Li A (2017). Development and clinical application of anti-HER2 monoclonal and bispecific antibodies for cancer treatment. Exp Hematol Oncol.

[7] Yu S, Li A, Liu Q, Yuan X, Xu H, Jiao D (2017). Recent advances of bispecific antibodies in solid tumors. J Hematol Oncol.

[8] Curran E, Stock W (2019). Taking a “BiTE out of ALL”: blinatumomab approval for MRD-positive ALL. Blood.

[9] Goebeler M-E, Bargou RC (2020). T cell-engaging therapies—BiTEs and beyond. Nat Rev Clin Oncol.

[10] Rader C (2020). Bispecific antibodies in cancer immunotherapy. Curr Opin Biotechnol.

[11] Anderson KG, Stromnes IM, Greenberg PD (2017). Obstacles posed by the tumor microenvironment to T cell activity: a case for synergistic therapies. Cancer Cell.

[12] Stamova S, Koristka S, Keil J, Arndt C, Feldmann A, Michalk I (2012). Cancer immunotherapy by retargeting of immune effector cells via recombinant bispecific antibody constructs. Antibodies.

[13] Kamperschroer C, Shenton J, Lebrec H, Leighton JK, Moore PA, Thomas O (2020). Summary of a workshop on preclinical and translational safety assessment of CD3 bispecifics. J Immunotoxicol.

[14] Brischwein K, Parr L, Pflanz S, Volkland J, Lumsden J, Klinger M (2007). Strictly target cell-dependent activation of T cells by bispecific single-chain antibody constructs of the BiTE class. J Immunother.

[15] Jen EY, Xu Q, Schetter A, Przepiorka D, Shen YL, Roscoe D (2019). FDA approval: blinatumomab for patients with B-cell precursor acute lymphoblastic leukemia in morphologic remission with minimal residual disease. Clin Cancer Res.

[16] Li H, Er Saw P, Song E (2020). Challenges and strategies for next-generation bispecific antibody-based antitumor therapeutics. Cell Mol Immunol.

[17] Kol A, Terwisscha van Scheltinga AG, Timmer-Bosscha H, Lamberts LE, Bensch F, de Vries EG (2014). HER3, serious partner in crime: therapeutic approaches and potential biomarkers for effect of HER3-targeting. Pharmacol Ther.

[18] Vidarsson G, Dekkers G, Rispens T (2014). IgG subclasses and allotypes: from structure to effector functions. Front Immunol.

[19] Weiner LM, Surana R, Wang S (2010). Monoclonal antibodies: versatile platforms for cancer immunotherapy. Nat Rev Immunol.

[20] Zhang X, Yang Y, Fan D, Xiong D (2017). The development of bispecific antibodies and their applications in tumor immune escape. Exp Hematol Oncol.

[21] Hosseini SS, Khalili S, Baradaran B, Bidar N, Shahbazi MA, Mosafer J (2021). Bispecific monoclonal antibodies for targeted immunotherapy of solid tumors: recent advances and clinical trials. Int J Biol Macromol.

[22] Labrijn AF, Janmaat ML, Reichert JM, Parren PWHI (2019). Bispecific antibodies: a mechanistic review of the pipeline. Nat Rev Drug Discov.

[23] Lameris R, de Bruin RC, Schneiders FL, en Henegouwen PMvB, Verheul HM, de Gruijl TD (2014). Bispecific antibody platforms for cancer immunotherapy. Crit Rev Oncol Hematol.

[24] Carter PJ (2006). Potent antibody therapeutics by design. Nat Rev Immunol.

[25] Kontermann RE, Brinkmann U (2015). Bispecific antibodies. Drug Discov Today.

[26] Wu C, Ying H, Grinnell C, Bryant S, Miller R, Clabbers A (2007). Simultaneous targeting of multiple disease mediators by a dual-variable-domain immunoglobulin. Nat Biotechnol.

[27] Krah S, Sellmann C, Rhiel L, Schröter C, Dickgiesser S, Beck J (2017). Engineering bispecific antibodies with defined chain pairing. N Biotechnol.

[28] Lejeune M, Köse MC, Duray E, Einsele H, Beguin Y, Caers J (2020). Bispecific, T-cell-recruiting antibodies in B-cell malignancies. Front Immunol.

[29] Kontermann RE (2011). Strategies for extended serum half-life of protein therapeutics. Curr Opin Biotechnol.

[30] Spiess C, Zhai Q, Carter PJ (2015). Alternative molecular formats and therapeutic applications for bispecific antibodies. Mol Immunol.

[31] Krishnamurthy A, Jimeno A (2018). Bispecific antibodies for cancer therapy: a review. Pharmacol Ther.

[32] Hirschhaeuser F, Walenta S, Mueller-Klieser W (2010). Efficacy of catumaxomab in tumor spheroid killing is mediated by its trifunctional mode of action. Cancer Immunol Immunother.

[33] Reichert JM (2014). Antibody Fc: linking adaptive and innate immunity. MAbs.

[34] Zhou Y, Zong H, Han L, Xie Y, Jiang H, Gilly J (2020). A novel bispecific antibody targeting CD3 and prolactin receptor (PRLR) against PRLR-expression breast cancer. J Exp Clin Cancer Res.

[35] Salnikov AV, Groth A, Apel A, Kallifatidis G, Beckermann BM, Khamidjanov A (2009). Targeting of cancer stem cell marker EpCAM by bispecific antibody EpCAMxCD3 inhibits pancreatic carcinoma. J Cell Mol Med.

[36] Maher J, Adami AA (2013). Antitumor immunity: easy as 1, 2, 3 with monoclonal bispecific trifunctional antibodies?. Cancer Res.

[37] Mølhøj M, Crommer S, Brischwein K, Rau D, Sriskandarajah M, Hoffmann P (2007). CD19-/CD3-bispecific antibody of the BiTE class is far superior to tandem diabody with respect to redirected tumor cell lysis. Mol Immunol.

[38] Zhang Z, Luo F, Cao J, Lu F, Zhang Y, Ma Y (2021). Anticancer bispecific antibody R&D advances: a study focusing on research trend worldwide and in China. J Hematol Oncol.

[39] Ishiguro T, Sano Y, Komatsu SI, Kamata-Sakurai M, Kaneko A, Kinoshita Y, et al. An anti-glypican 3/CD3 bispecific T cell-redirecting antibody for treatment of solid tumors. Sci Transl Med. 2017; 9(410). 10.1126/scitranslmed.aal4291 28978751

[40] Gaber A, Lenarčič B, Pavšič M (2020). Current view on EpCAM structural biology. Cells.

[41] Gires O, Pan M, Schinke H, Canis M, Baeuerle PA (2020). Expression and function of epithelial cell adhesion molecule EpCAM: where are we after 40 years?. Cancer Metastasis Rev.

[42] Fagotto F (2020). EpCAM as modulator of tissue plasticity. Cells.

[43] Went P, Vasei M, Bubendorf L, Terracciano L, Tornillo L, Riede U (2006). Frequent high-level expression of the immunotherapeutic target Ep-CAM in colon, stomach, prostate and lung cancers. Br J Cancer.

[44] Linke R, Klein A, Seimetz D (2010). Catumaxomab: clinical development and future directions. MAbs.

[45] Kubo M, Umebayashi M, Kurata K, Mori H, Kai M, Onishi H (2018). Catumaxomab with activated T-cells efficiently lyses chemoresistant EpCAM-positive triple-negative breast cancer cell lines. Anticancer Res.

[46] Heiss MM, Ströhlein MA, Jäger M, Kimmig R, Burges A, Schoberth A (2005). Immunotherapy of malignant ascites with trifunctional antibodies. Int J Cancer.

[47] Belau A, Pfisterer J, Wimberger P, Kurzeder C, Du Bois A, Sehouli J (2007). Randomized, multicenter, two-dose level, open-label, phase IIa study with the intraperitoneally infused trifunctional bispecific antibody catumaxomab (anti-EpCAM× anti-CD3) to select the better dose level in platinum refractory epithelial ovarian cancer patients. J Clin Oncol.

[48] Heiss MM, Murawa P, Koralewski P, Kutarska E, Kolesnik OO, Ivanchenko VV (2010). The trifunctional antibody catumaxomab for the treatment of malignant ascites due to epithelial cancer: results of a prospective randomized phase II/III trial. Int J Cancer.

[49] Sebastian M, Passlick B, Friccius-Quecke H, Jäger M, Lindhofer H, Kanniess F (2007). Treatment of non-small cell lung cancer patients with the trifunctional monoclonal antibody catumaxomab (anti-EpCAM× anti-CD3): a phase I study. Cancer Immunol Immunother.

[50] Mau-Sørensen M, Dittrich C, Dienstmann R, Lassen U, Büchler W, Martinius H (2015). A phase I trial of intravenous catumaxomab: a bispecific monoclonal antibody targeting EpCAM and the T cell coreceptor CD3. Cancer Chemother Pharmacol.

[51] Knödler M, Körfer J, Kunzmann V, Trojan J, Daum S, Schenk M (2018). Randomised phase II trial to investigate catumaxomab (anti-EpCAM × anti-CD3) for treatment of peritoneal carcinomatosis in patients with gastric cancer. Br J Cancer.

[52] Brischwein K, Schlereth B, Guller B, Steiger C, Wolf A, Lutterbuese R (2006). MT110: a novel bispecific single-chain antibody construct with high efficacy in eradicating established tumors. Mol Immunol.

[53] Schlereth B, Fichtner I, Lorenczewski G, Kleindienst P, Brischwein K, da Silva A (2005). Eradication of tumors from a human colon cancer cell line and from ovarian cancer metastases in immunodeficient mice by a single-chain Ep-CAM-/CD3-bispecific antibody construct. Cancer Res.

[54] Amann M, Brischwein K, Lutterbuese P, Parr L, Petersen L, Lorenczewski G (2008). Therapeutic window of MuS110, a single-chain antibody construct bispecific for murine EpCAM and murine CD3. Cancer Res.

[55] Kebenko M, Goebeler ME, Wolf M, Hasenburg A, Seggewiss-Bernhardt R, Ritter B (2018). A multicenter phase 1 study of solitomab (MT110, AMG 110), a bispecific EpCAM/CD3 T-cell engager (BiTE®) antibody construct, in patients with refractory solid tumors. Oncoimmunology..

[56] Beauchemin N, Arabzadeh A (2013). Carcinoembryonic antigen-related cell adhesion molecules (CEACAMs) in cancer progression and metastasis. Cancer Metastasis Rev.

[57] Blumenthal RD, Hansen HJ, Goldenberg DM (2005). Inhibition of adhesion, invasion, and metastasis by antibodies targeting CEACAM6 (NCA-90) and CEACAM5 (Carcinoembryonic Antigen). Cancer Res.

[58] You W, Yan L, Cai Z, Xie L, Sheng N, Wang G (2020). Clinical significances of positive postoperative serum CEA and post-preoperative CEA increment in stage II and III colorectal cancer: a multicenter retrospective study. Front Oncol.

[59] Nakamura H, Nishimura T (2017). History, molecular features, and clinical importance of conventional serum biomarkers in lung cancer. Surg Today.

[60] Grunnet M, Sorensen JB (2012). Carcinoembryonic antigen (CEA) as tumor marker in lung cancer. Lung Cancer.

[61] Peng L, Oberst MD, Huang J, Brohawn P, Morehouse C, Lekstrom K (2012). The CEA/CD3-bispecific antibody MEDI-565 (MT111) binds a nonlinear epitope in the full-length but not a short splice variant of CEA. PLoS ONE.

[62] Oberst MD, Fuhrmann S, Mulgrew K, Amann M, Cheng L, Lutterbuese P (2014). CEA/CD3 bispecific antibody MEDI-565/AMG 211 activation of T cells and subsequent killing of human tumors is independent of mutations commonly found in colorectal adenocarcinomas. MAbs.

[63] Moek KL, Waaijer SJH, Kok IC, Suurs FV, Brouwers AH, Menke-van der Houven van Oordt CW (2019). (89)Zr-labeled bispecific T-cell engager AMG 211 PET shows AMG 211 accumulation in CD3-rich tissues and clear heterogeneous tumor uptake. Clin Cancer Res.

[64] Pishvaian M, Morse MA, McDevitt J, Norton JD, Ren S, Robbie GJ (2016). Phase 1 dose escalation study of MEDI-565, a bispecific T-cell engager that targets human carcinoembryonic antigen, in patients with advanced gastrointestinal adenocarcinomas. Clin Colorectal Cancer.

[65] Bacac M, Fauti T, Sam J, Colombetti S, Weinzierl T, Ouaret D (2016). A novel carcinoembryonic antigen T-cell bispecific antibody (CEA TCB) for the treatment of solid tumors. Clin Cancer Res.

[66] Lehmann S, Perera R, Grimm H-P, Sam J, Colombetti S, Fauti T (2016). In vivo fluorescence imaging of the activity of CEA TCB, a novel T-cell bispecific antibody, reveals highly specific tumor targeting and fast induction of T-cell–mediated tumor killing. Clin Cancer Res.

[67] Tabernero J, Melero I, Ros W, Argiles G, Marabelle A, Rodriguez-Ruiz ME (2017). Phase Ia and Ib studies of the novel carcinoembryonic antigen (CEA) T-cell bispecific (CEA CD3 TCB) antibody as a single agent and in combination with atezolizumab: Preliminary efficacy and safety in patients with metastatic colorectal cancer (mCRC). J Clin Oncol.

[68] Haberkorn U, Eder M, Kopka K, Babich JW, Eisenhut M (2016). New strategies in prostate cancer: prostate-specific membrane antigen (PSMA) ligands for diagnosis and therapy. Clin Cancer Res.

[69] Perner S, Hofer MD, Kim R, Shah RB, Li H, Möller P (2007). Prostate-specific membrane antigen expression as a predictor of prostate cancer progression. Hum Pathol.

[70] Mannweiler S, Amersdorfer P, Trajanoski S, Terrett JA, King D, Mehes G (2009). Heterogeneity of prostate-specific membrane antigen (PSMA) expression in prostate carcinoma with distant metastasis. Pathol Oncol Res.

[71] Bostwick DG, Pacelli A, Blute M, Roche P, Murphy GP (1998). Prostate specific membrane antigen expression in prostatic intraepithelial neoplasia and adenocarcinoma: a study of 184 cases. Cancer.

[72] Ross JS, Sheehan CE, Fisher HA, Kaufman RP, Kaur P, Gray K (2003). Correlation of primary tumor prostate-specific membrane antigen expression with disease recurrence in prostate cancer. Clin Cancer Res.

[73] Barrio M, Fendler WP, Czernin J, Herrmann K (2016). Prostate specific membrane antigen (PSMA) ligands for diagnosis and therapy of prostate cancer. Expert Rev Mol Diagn.

[74] Hummel HD, Kufer P, Grüllich C, Seggewiss-Bernhardt R, Deschler-Baier B, Chatterjee M (2021). Pasotuxizumab, a BiTE(®) immune therapy for castration-resistant prostate cancer: phase I, dose-escalation study findings. Immunotherapy.

[75] Hummel H-D, Kufer P, Grüllich C, Deschler-Baier B, Chatterjee M, Goebeler M-E (2019). Phase 1 study of pasotuxizumab (BAY 2010112), a PSMA-targeting Bispecific T cell Engager (BiTE) immunotherapy for metastatic castration-resistant prostate cancer (mCRPC). J Clin Oncol.

[76] Bendell JC, Fong L, Stein MN, Beer TM, Ross A, Gao X (2020). First-in-human phase I study of HPN424, a tri-specific half-life extended PSMA-targeting T-cell engager in patients with metastatic castration-resistant prostate cancer (mCRPC). J Clin Oncol.

[77] Nauseef JT, Bander NH, Tagawa ST (2021). Emerging prostate-specific membrane antigen-based therapeutics: small molecules, antibodies, and beyond. Eur Urol Focus.

[78] Deegen P, Thomas O, Nolan-Stevaux O, Li S, Wahl J, Bogner P (2021). The PSMA-targeting half-life extended BiTE therapy AMG 160 has potent antitumor activity in preclinical models of metastatic castration-resistant prostate cancer. Clin Cancer Res.

[79] Tran B, Horvath L, Dorff T, Rettig M, Lolkema M, Machiels J (2020). 609O Results from a phase I study of AMG 160, a half-life extended (HLE), PSMA-targeted, bispecific T-cell engager (BiTE®) immune therapy for metastatic castration-resistant prostate cancer (mCRPC). Ann Oncol.

[80] Roskoski R (2019). Small molecule inhibitors targeting the EGFR/ErbB family of protein-tyrosine kinases in human cancers. Pharmacol Res.

[81] Saxena R, Dwivedi A (2012). ErbB family receptor inhibitors as therapeutic agents in breast cancer: current status and future clinical perspective. Med Res Rev.

[82] Tebbutt N, Pedersen MW, Johns TG (2013). Targeting the ERBB family in cancer: couples therapy. Nat Rev Cancer.

[83] Lopez-Albaitero A, Xu H, Guo H, Wang L, Wu Z, Tran H (2017). Overcoming resistance to HER2-targeted therapy with a novel HER2/CD3 bispecific antibody. Oncoimmunology..

[84] Jones KL, Buzdar AU (2009). Evolving novel anti-HER2 strategies. Lancet Oncol.

[85] Jäger M, Schoberth A, Ruf P, Hess J, Lindhofer H (2009). The trifunctional antibody ertumaxomab destroys tumor cells that express low levels of human epidermal growth factor receptor 2. Cancer Res.

[86] Kumagai S, Koyama S, Nishikawa H (2021). Antitumour immunity regulated by aberrant ERBB family signalling. Nat Rev Cancer.

[87] Kiewe P, Hasmüller S, Kahlert S, Heinrigs M, Rack B, Marmé A (2006). Phase I trial of the trifunctional anti-HER2 × anti-CD3 antibody ertumaxomab in metastatic breast cancer. Clin Cancer Res.

[88] Haense N, Atmaca A, Pauligk C, Steinmetz K, Marmé F, Haag GM (2016). A phase I trial of the trifunctional anti Her2 × anti CD3 antibody ertumaxomab in patients with advanced solid tumors. BMC Cancer.

[89] Nyati MK, Morgan MA, Feng FY, Lawrence TS (2006). Integration of EGFR inhibitors with radiochemotherapy. Nat Rev Cancer.

[90] Roskoski RJPr (2014). The ErbB/HER family of protein-tyrosine kinases and cancer. Pharmacol Res.

[91] Huang L, Jiang S, Shi Y (2020). Tyrosine kinase inhibitors for solid tumors in the past 20 years (2001–2020). J Hematol Oncol.

[92] Wang Q, Yang S, Wang K, Sun SY (2019). MET inhibitors for targeted therapy of EGFR TKI-resistant lung cancer. J Hematol Oncol.

[93] Neijssen J, Cardoso RMF, Chevalier KM, Wiegman L, Valerius T, Anderson GM (2021). Discovery of amivantamab (JNJ-61186372), a bispecific antibody targeting EGFR and MET. J Biol Chem.

[94] Yun J, Lee SH, Kim SY, Jeong SY, Kim JH, Pyo KH (2020). Antitumor activity of amivantamab (JNJ-61186372), an EGFR-MET bispecific antibody, in diverse models of EGFR exon 20 insertion-driven NSCLC. Cancer Discov.

[95] Vijayaraghavan S, Lipfert L, Chevalier K, Bushey BS, Henley B, Lenhart R (2020). Amivantamab (JNJ-61186372), an Fc enhanced EGFR/cMet bispecific antibody, induces receptor downmodulation and antitumor activity by monocyte/macrophage trogocytosis. Mol Cancer Ther.

[96] Moores SL, Chiu ML, Bushey BS, Chevalier K, Luistro L, Dorn K (2016). A novel bispecific antibody targeting EGFR and cMet is effective against EGFR inhibitor-resistant lung tumors. Cancer Res.

[97] Haura EB, Cho BC, Lee JS, Han J-Y, Lee KH, Sanborn RE (2019). JNJ-61186372 (JNJ-372), an EGFR-cMet bispecific antibody, in EGFR-driven advanced non-small cell lung cancer (NSCLC). J Clin Oncol.

[98] Park K, John T, Kim S-W, Lee JS, Shu CA, Kim D-W (2020). Amivantamab (JNJ-61186372), an anti-EGFR-MET bispecific antibody, in patients with EGFR exon 20 insertion (exon20ins)-mutated non-small cell lung cancer (NSCLC). J Clin Oncol.

[99] Remon J, Hendriks LEL, Cardona AF, Besse B (2020). EGFR exon 20 insertions in advanced non-small cell lung cancer: a new history begins. Cancer Treat Rev.

[100] McDonagh CF, Huhalov A, Harms BD, Adams S, Paragas V, Oyama S (2012). Antitumor activity of a novel bispecific antibody that targets the ErbB2/ErbB3 oncogenic unit and inhibits heregulin-induced activation of ErbB3. Mol Cancer Ther.

[101] Zhang B, Lahdenranta J, Du J, Kirouac D, Nguyen S, Overland R (2013). Abstract 4633: MM-111, a bispecific HER2 and HER3 antibody, synergistically combines with trastuzumab and paclitaxel in preclinical models of gastric cancer. Cancer Res.

[102] Drilon A, Somwar R, Mangatt BP, Edgren H, Desmeules P, Ruusulehto A (2018). Response to ERBB3-directed targeted therapy in NRG1-rearranged cancers. Cancer Discov.

[103] de Vries Schultink AHM, Doornbos RP, Bakker ABH, Bol K, Throsby M, Geuijen C (2018). Translational PK-PD modeling analysis of MCLA-128, a HER2/HER3 bispecific monoclonal antibody, to predict clinical efficacious exposure and dose. Invest New Drugs.

[104] Li N, Gao W, Zhang Y-F, Ho M (2018). Glypicans as cancer therapeutic targets. Trends Cancer.

[105] McGlynn KA, Petrick JL, El-Serag HB (2021). Epidemiology of hepatocellular carcinoma. Hepatology.

[106] Shirakawa H, Suzuki H, Shimomura M, Kojima M, Gotohda N, Takahashi S (2009). Glypican-3 expression is correlated with poor prognosis in hepatocellular carcinoma. Cancer Sci.

[107] Ning S, Bin C, Na H, Peng S, Yi D, Xiang-hua Y (2012). Glypican-3, a novel prognostic marker of hepatocellular cancer, is related with postoperative metastasis and recurrence in hepatocellular cancer patients. Mol Biol Rep.

[108] Cully M (2017). Bispecific antibody directs T cells to solid tumours. Nat Rev Drug Discov.

[109] Yu L, Yang X, Huang N, Lang Q-L, He Q-L, Wang J-H (2020). A novel targeted GPC3/CD3 bispecific antibody for the treatment hepatocellular carcinoma. Cancer Biol Ther.

[110] Kotanides H, Li Y, Malabunga M, Carpenito C, Eastman SW, Shen Y (2020). Bispecific targeting of PD-1 and PD-L1 enhances T-cell activation and antitumor immunity. Cancer Immunol Res.

[111] Hellmann MD, Bivi N, Calderon B, Shimizu T, Delafontaine B, Liu ZT (2021). Safety and immunogenicity of LY3415244, a bispecific antibody against TIM-3 and PD-L1, in patients with advanced solid tumors. Clin Cancer Res.

[112] Yi M, Zhang J, Li A, Niu M, Yan Y, Jiao Y (2021). The construction, expression, and enhanced anti-tumor activity of YM101: a bispecific antibody simultaneously targeting TGF-β and PD-L1. J Hematol Oncol.

[113] Lan Y, Zhang D, Xu C, Hance KW, Marelli B, Qi J, et al. Enhanced preclinical antitumor activity of M7824, a bifunctional fusion protein simultaneously targeting PD-L1 and TGF-β. Sci Transl Med. 2018; 10(424). 10.1126/scitranslmed.aan5488 29343622

[114] Yi M, Niu M, Zhang J, Li S, Zhu S, Yan Y (2021). Combine and conquer: manganese synergizing anti-TGF-β/PD-L1 bispecific antibody YM101 to overcome immunotherapy resistance in non-inflamed cancers. J Hematol Oncol.

[115] Chung AS, Lee J, Ferrara N (2010). Targeting the tumour vasculature: insights from physiological angiogenesis. Nat Rev Cancer.

[116] Huang J, Hu W, Hu L, Previs RA, Dalton HJ, Yang X-Y (2016). Dll4 inhibition plus aflibercept markedly reduces ovarian tumor growth. Mol Cancer Ther.

[117] Jimeno A, Moore KN, Gordon M, Chugh R, Diamond JR, Aljumaily R (2019). A first-in-human phase 1a study of the bispecific anti-DLL4/anti-VEGF antibody navicixizumab (OMP-305B83) in patients with previously treated solid tumors. Invest New Drugs.

[118] Perez-Fidalgo JA, Ortega B, Simon S, Samartzis EP, Boussios S (2020). NOTCH signalling in ovarian cancer angiogenesis. Ann Transl Med.

[119] Nathan P, Hassel JC, Rutkowski P, Baurain JF, Butler MO, Schlaak M (2021). Overall survival benefit with tebentafusp in metastatic uveal melanoma. N Engl J Med.

[120] Moore PA, Shah K, Yang Y, Alderson R, Roberts P, Long V (2018). Development of MGD007, a gpA33 x CD3-bispecific DART protein for T-cell immunotherapy of metastatic colorectal cancer. Mol Cancer Ther.

[121] von Stackelberg A, Locatelli F, Zugmaier G, Handgretinger R, Trippett TM, Rizzari C (2016). Phase I/Phase II study of blinatumomab in pediatric patients with relapsed/refractory acute lymphoblastic leukemia. J Clin Oncol.

[122] Topp MS, Gökbuget N, Stein AS, Zugmaier G, O'Brien S, Bargou RC (2015). Safety and activity of blinatumomab for adult patients with relapsed or refractory B-precursor acute lymphoblastic leukaemia: a multicentre, single-arm, phase 2 study. Lancet Oncol.

[123] Shimabukuro-Vornhagen A, Gödel P, Subklewe M, Stemmler HJ, Schlößer HA, Schlaak M (2018). Cytokine release syndrome. J Immunother Cancer.

[124] Klinger M, Brandl C, Zugmaier G, Hijazi Y, Bargou RC, Topp MS (2012). Immunopharmacologic response of patients with B-lineage acute lymphoblastic leukemia to continuous infusion of T cell–engaging CD19/CD3-bispecific BiTE antibody blinatumomab. Blood.

[125] Iwata Y, Sasaki M, Harada A, Taketo J, Hara T, Akai S (2019). Daily ascending dosing in cynomolgus monkeys to mitigate cytokine release syndrome induced by ERY22, surrogate for T-cell redirecting bispecific antibody ERY974 for cancer immunotherapy. Toxicol Appl Pharmacol.

[126] Lee DW, Gardner R, Porter DL, Louis CU, Ahmed N, Jensen M (2014). Current concepts in the diagnosis and management of cytokine release syndrome. Blood.

[127] Nishimoto N, Kishimoto T (2006). Interleukin 6: from bench to bedside. Nat Clin Pract Rheumatol.

[128] Teachey DT, Rheingold SR, Maude SL, Zugmaier G, Barrett DM, Seif AE (2013). Cytokine release syndrome after blinatumomab treatment related to abnormal macrophage activation and ameliorated with cytokine-directed therapy. Blood.

